# A method to prevent clogging and clustering in microfluidic systems using microbubble streaming

**DOI:** 10.1063/5.0214436

**Published:** 2024-07-02

**Authors:** Amirabas Bakhtiari, Christian J. Kähler

**Affiliations:** Institute for Fluid Mechanics and Aerodynamics, Bundeswehr University Munich, Werner-Heisenberg-Weg 39, 85579 Neubiberg, Germany

## Abstract

This paper presents an innovative strategy to address the issues of clogging and cluster-related challenges in microchannels within microfluidic devices. Leveraging three-dimensional (3D) microbubble streaming as a dynamic solution, our approach involves the controlled activation of microbubbles near channel constrictions, inducing microstreaming with distinctive features. This microstreaming, characterized by a high non-uniform 3D gradient and significant shear stress, effectively inhibits arch formation at constrictions and disintegrates particle clusters, demonstrating real-time prevention of clogging incidents and blockages. This study includes experimental validation of the anti-clogging technique, a detailed examination of microstreaming phenomena, and their effects on clogging and clustering issues. It also incorporates statistical analyses performed in various scenarios to verify the method’s effectiveness and adaptability. Moreover, a versatile control system has been designed that operates in event-triggered, continuous, or periodic modes, which suits different lab-on-a-chip applications and improves the overall functionality of microfluidic systems.

## I. INTRODUCTION

Experience shows that solid particles passed through a relatively narrow constriction can be disrupted by the formation of clogs that block the constriction.^1^ Clogging occurs in various systems where particles are retained or discharged, e.g., in a hungry flock entering a room through a door,^1,2^ in crowds of people evacuating a room in a life-threatening situation,^3,4^ in the emptying of dry silos,^5,6^ in the neck of an hourglass,^7^ or in microfluidic and filtration circuits, where a loaded fluid enters a device or passes through a membrane.^8–10^ However, contrary to the apparent similarities, different clogging mechanisms prevail in these systems. In hourglasses, for example, dry granules clog when a stable static particle arch blocks the constriction,^11^ independent of flow velocity^12^ and almost independent of the constriction angle,^11^ whereas in colloidal systems, other clogging mechanisms often occur, involving single particle effects due to wall–particle adhesion or particle aggregation.^10,13,14^ As a result, many microfluidic channels are typically designed as single-use devices for short-term use because the microchannels become clogged with biological cells, drastically limiting their use in continuous systems.^15^ Similar to many biological systems, suspensions of microparticles play a particularly important role in many microfluidic applications, as the liquid phase enables the transport of solids,^16–21^ the formation of blockages dramatically affects the performance of these systems and effectively limits the use of microfluidic technology. In recent years, efforts have been made to address the challenge of clogging during blood plasma separation processes by employing dielectrophoresis, as documented in various studies.^15,22–25^ These endeavors underscore the significance of anti-clogging techniques, especially in lab-on-a-chip applications characterized by extended operating times in continuous systems. Although dielectrophoresis offers various advantages, its application can entail certain drawbacks depending on the context. These drawbacks may include the risk of cell damage, restricted applicability, electrode fouling, complexity, cost implications, and maintenance challenges.^26–28^ To address these concerns, recirculating flows offer distinct advantages over directed motion strategies like dielectrophoresis. In passive microfluidic systems that do not require external power, devices can be designed with loops or spiral channels to naturally induce recirculating flows, keeping particles suspended and preventing sedimentation or adhesion to the walls.^29^ Similarly, curved microchannels create helical streamlines, resulting in Dean flow, where larger particles experience a lift force that moves them toward the capture zone, while smaller particles pass through a bypass.^30^ Another technique involves lateral flow microfluidic sieving, where fluid with mixed particles flows through a filter. Gentle vibrations prevent clogging and ensure continuous operation.^31^ Researchers have also found that optimizing the frequency and amplitude of pulsatile flows can significantly delay microchannel clogging compared to steady flow conditions and is more effective than flow reversal.^32^ While passive methods or alternating and reversal flow can mitigate clogging in specific applications, their effectiveness compared to active methods that use strong flow to break down clusters remains unproven. Additionally, complex microfluidic systems may present challenges in fabrication, operation, and maintenance. An alternative approach involves the cost-effective utilization of microbubble streaming, known for its inherent biocompatibility.^33^ When a microchannel containing a microbubble is stimulated by a piezotransducer, particularly near the bubble’s resonant frequency, it induces primary oscillatory fluid motion. This primary flow, interacting with the channel walls, generates a secondary flow pattern marked by the development of 3D counter-rotating vortices. This microstreaming phenomenon can be precisely tuned across a range of resonant frequencies by adjusting the amplitudes of actuation.^34–37^

Over the past decade, microbubble streaming has emerged as a non-invasive and biocompatible tool in various advanced lab-on-a-chip devices. These devices have showcased their versatility by enabling functions such as particle sorting, mixing, focusing, positioning, and size-selective separation of both microparticles and biological cells.^36,38–41^ This method now holds promise as a solution to universally address clogging issues in lab-on-a-chip and microfluidics applications, providing a balance between simplicity, efficacy, and practicality.

In the present study, we introduce an innovative anti-clogging method employing microbubble streaming to enhance the real-time and continuous microfluidic operations of lab-on-a-chip devices. Our approach harnesses microbubble streaming in the vicinity of constrictions and illustrates multiple operational strategies (event-triggered, continuous, and periodic activation of microbubble streaming) to prevent clogging incidents or alleviate blockages as they occur. To achieve this, we incorporate a microbubble-containing cavity adjacent to the constriction, activated by a piezotransducer affixed to the microchip, which generates 3D counter-rotating vortices within this region. Microstreaming phenomena generate unique characteristics that, when considered individually or in conjunction with one another (depending on factors such as particle sizes, operational modes, and flow conditions), have the potential to mitigate or address clogging issues within microchannels. Our research validates our method through experimental tests and a detailed study of these occurrences, augmented by thorough statistical analyses that cover a variety of scenarios, such as different particle sizes and operational modes. Significantly, our design and concept stand out for their simplicity and do not require moving parts, depending instead on bubble oscillation. This inherent robustness obviates the need for special maintenance or cleaning, rendering our microchip easily integrated into diverse microfluidic applications. Furthermore, we have developed a control system that can operate in continuous, periodic, or event-triggered modes, offering flexibility to accommodate diverse applications with specific needs, even when continuous microstreaming is not beneficial.

## II. EXPERIMENTAL SETUP

In this section, we describe the experimental design shown in Fig. 2. Our experimental system generally consists of a microfluidic system, an optical setup, and the control system mounted on a vibration-damped stage to minimize experimental errors.

### A. Preparation of samples

This research utilized polystyrene particles with red fluorescence (PS-FluoRed: excitation/emission 530/607 nm) in different sizes of 50 and 100 μm with neck-to-particle size ratios (*W/d*) of 3 and 1.5, respectively. These particles were sourced from Microparticles GmbH, Germany. These particles are stabilized with negatively charged sulfate groups. The negative charge, attributed to sulfate groups on the microsphere surface, played a crucial role in preventing agglomeration and adhesion. This charge-induced repulsion among particles facilitated dispersion, hindering cluster formation, and minimized adhesion to the polydimethylsiloxane (PDMS) channel walls, ensuring unimpeded particle movement. The negative charge also contributed to the overall stability of the microspheres in the aqueous solution, enhancing reproducibility and control of accumulation patterns.^42^ In the sample preparation process, microspheres were immersed in an aqueous solution containing 23.8 w – w% glycerol.^43,44^ The objective was to attain neutral buoyancy for the particles, aligning their density with that of the surrounding medium. To ensure even dispersion, rigorous mixing of the microspheres was undertaken, a crucial step for promoting random and consistent particle entry. It is noteworthy, however, that achieving precise homogeneity in particle concentration within the channel proved challenging due to recurrent clogging issues. Consequently, an extended backflow period was deemed necessary to effectively homogenize particle distribution in the solution, as detailed in the Results section.

### B. Microfluidic system configuration

**FIG. 1.**
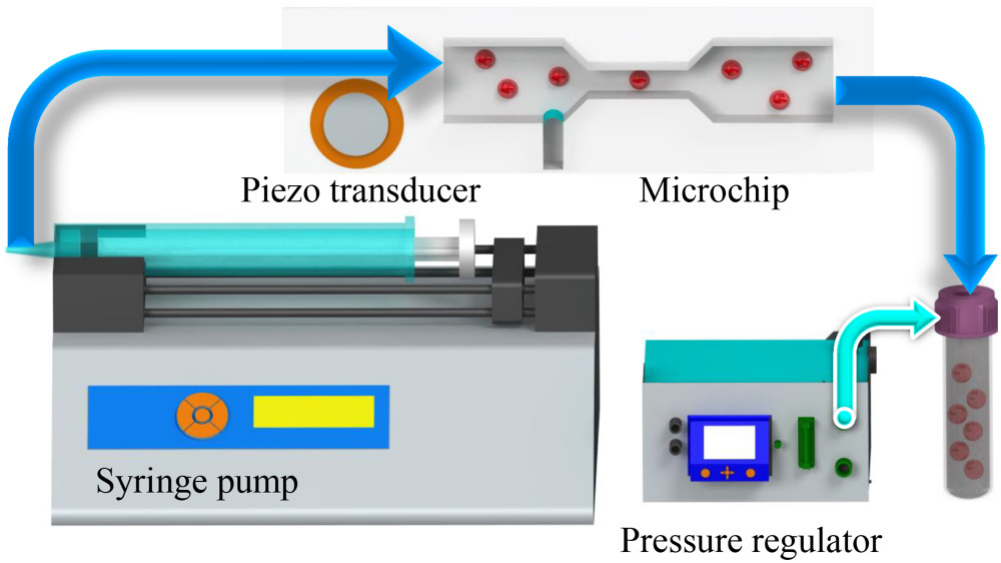
The microfluidic chip and its associated flow control system. The 20 mm microchannel showcases a rectangular cross section (*H* = 150 μm × *W* = 450 μm), transitioning to a smaller square cross section (*H* = 150 μm × *W* = 150 μm) through a linear narrowing with a $45^{{}^{\circ}}$ angle, forming a two-dimensional nozzle converging toward the constriction. At the nozzle’s onset, there is a cavity with a width of *w* = 80 μm and a length of *h* = 500 μm. The microbubble is stimulated by a piezoelectric transducer, while the syringe pump regulates the flow rate, and a pressure regulator maintains liquid and bubble pressure for size stabilization.

The microfluidic setup, illustrated in Fig. 1, encompasses transparent microchannels and integrated flow control systems. To create the anti-clogging microchannel, we employed the conventional soft lithography technique, adapting the methodology detailed by Wang *et al.*^45^ However, we utilized the projection photolithography method suggested by Ostmann *et al.*^46^ This method employs a negative magnification technique, enabling the use of cost-effective masks created by standard printers with lower printing quality for the soft lithography fabrication process. The 20 mm straight microchannel features a rectangular cross section (*H* = 150 μm × *W* = 450 μm), transitioning to a smaller square cross section (*H* = 150 μm × *W* = 150 μm) through a linear narrowing of the microchannel with an angle of $45^{{}^{\circ}}$, forming a two-dimensional nozzle converging toward the constriction. At the nozzle’s initiation, a cavity with a width of *w* = 80 μm and a length of *h* = 500 μm is situated. Based on the findings of previous research,^34–36,47,48^ the width of the microbubble cavity was carefully selected. It was important to ensure that the cavity was not excessively wide, which could lead to the detachment of the bubble, nor excessively narrow, which might result in the formation of diminutive bubbles generating only weak streaming with negligible effects on the flow field. Additionally, the decision to use a single bubble near the throat was based on a preliminary systematic investigation into the channel design and microbubble configurations. This study considered both the location and number of bubbles to optimize performance. The results of this optimization study are provided in the supplementary data (Fig. S1 in the supplementary material).

Upon the swift infusion of liquid into the microchannel, a gas (in this case, air) pocket becomes confined within the lateral cavity, giving rise to the formation of a quasi-cylindrical microbubble. It is essential to highlight that the composition of the bubble need not be limited to air; alternatively, different gases, such as argon, can be employed. This is particularly relevant in instances where the conveyed fluids and particles must avoid interaction with atmospheric air. When a microbubble is subjected to acoustic excitation, its interface oscillates at much higher amplitudes than the incoming sound wave due to the high compressibility of the gas inside. This initially creates an oscillatory flow around the bubble at the same frequency as the acoustic actuation. In the presence of walls, this oscillatory flow generates a secondary steady streaming flow in the form of counter-rotating vortices. While microstreaming can occur at the resonant frequencies of the microbubble, it can also occur at non-resonant frequencies due to non-linear effects, harmonic generation, and other factors.^49–51^ Ensuring experimental reproducibility, microbubble size consistency is maintained by controlling the pressure differential between the channel’s interior and the surrounding ambient pressure (approximately 6–8 mbar pressure difference in this study), following the approach proposed by Volk *et al.*^52^ This control is achieved through the regulation of the aqueous sample solution’s flow into the channel, facilitated by a syringe pump (neMESYS, Germany) and a pressure regulator (FluigentMFCS^TM^-EZ, 0–1000 mbar, France), as illustrated in Fig. 1. It is also worth noting that although we did not observe any collapse of the microbubble under the tested conditions, we acknowledge that clogging can occur when the bubble is not excited by the piezoelectric element (i.e., when the bubble is in the off mode). In such cases, if the fluid flow is driven by a syringe pump instead of a pressure controller, increased back pressure could cause bubble collapse and particle penetration into the cavity. To recover from such events without resetting the entire system, we propose a design modification by connecting the end of the cavity directly to a pressure controller. This would allow for flushing the cavity by applying pressure, thereby removing the particles and regenerating a new bubble, as well as enabling precise control of the bubble level. This approach has been successfully implemented in our previous research and can be readily applied here as well. Further details can be found in the work by Bakhtiari *et al.*^53^

### C. Optical setup

The optical configuration includes an upright Zeiss AxioImager.Z2 microscope with dichroic filter, a 10 × objective (EC Plan Neouar 10 ×/0.3 M27), and an sCMOS camera (pco.edge 5.5), which is suitable for both brightfield and darkfield imaging. In bright-field microscopy, the camera records the shadows of the particles when they are illuminated from behind. In epifluorescence microscopy, fluorescent particles are illuminated with a continuous wave laser (CW laser) or a high-power light-emitting diode (LED), and the emitted light is recorded by the camera.

While both bright and dark field modes are possible in our control system, dark field imaging was chosen for Micro-Particle Tracking Velocimetry (microPTV) with 2 μm tracer particles. This choice, which is justified by a high signal-to-noise ratio^54^ and effective filtering of unwanted features such as impurities in the fluid or channel contamination during imaging, significantly reduces uncertainties in flow characterization. Conversely, in the context of our anti-clogging experiments, larger particles (50 and 100 μm) were employed. The utilization of these larger particles eliminated concerns related to the signal-to-noise ratio. Therefore, bright-field imaging was selected as the preferred method for visualization due to its enhanced capability to depict the data accurately.

### D. Control system setup

**FIG. 2.**
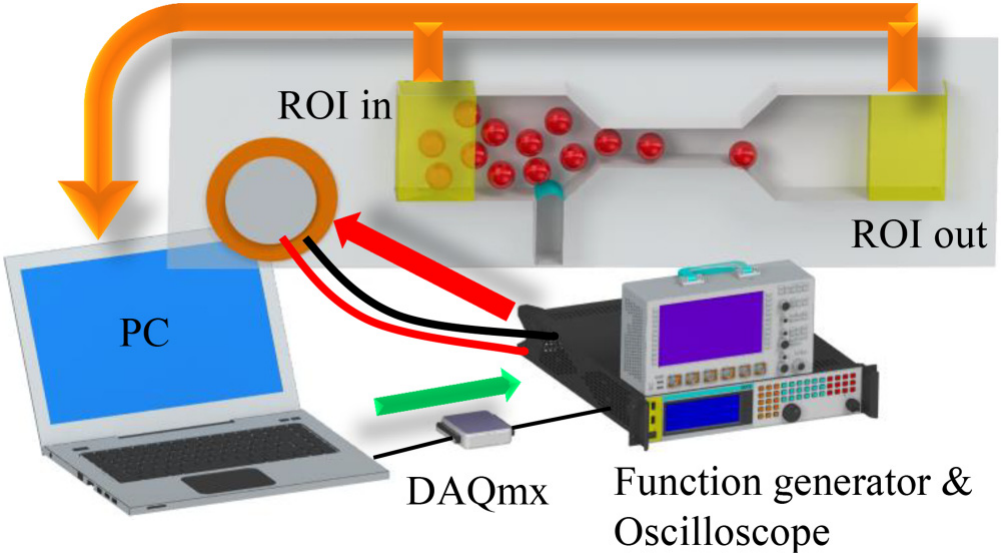
The customized LabVIEW control system oversees a range of operations, such as particle counting at the microchannel’s inlet and outlet, piezotransducer control, pressure regulation, and flow rate management. The highlighted yellow regions of interest (ROIs) are designated for particle counting, and the subtraction of their results is utilized to detect potential clogging events.

The operational control of the anti-clogging system is facilitated by LabVIEW (National Instrument, USA). LabVIEW oversees the entire process, including real-time image acquisition, image analysis, and clog detection during live operation. The identification of clogs occurs when the system experiences a substantial decrease in the count of detected escaped particles compared to the incoming particle count. For the transmission of the predetermined electrical signal (tuned to the resonant frequency of the microbubble) to the piezoelectric transducer on the microfluidic chip, a function generator (GW INSTEK AFG-2225), an amplifier (Krohn Hite 7500), and an oscilloscope (Teledyne LeCroy HDO6104) are employed. The function generator is synchronized with a National Instruments USB-6002  DAQmx data acquisition device, which is, in turn, orchestrated by LabVIEW. This arrangement allows the triggering of the function generator at specified intervals, enabling continuous, periodic, or time-specific excitation of the piezoelectric transducer during the event-triggered mode (see Fig. 2).

## III. METHODS

The anti-clogging concept operates on the principle of introducing the fast-3D-flow perturbation of microbubble streaming just prior to a constriction point, which is the location with the highest likelihood of clogging occurrence. Microstreaming phenomena produce distinct attributes that, when examined independently or in combination, offer the potential to alleviate or resolve clogging problems within microchannels. We will now highlight some of the key aspects in this regard.

### A. Mitigating arch formation in microfluidic systems

In the realm of microfluidics, where laminar flow is prevalent, the primary cause of clogging in constrictions and microchannels, especially when dealing with non-adherent and individually dispersed microparticles (with no particle clusters), is the creation of bridge-like formations known as “arches.” These arches come into existence when a critical number of particles converge at the constriction within a brief timeframe and align themselves in a manner that collectively spans the channel, resulting in a stable and obstructive structure. These arch structures can spontaneously materialize, effectively impeding the flow of additional particles downstream.^42^ Our approach aims to counteract the formation of these arches or even dislodge existing ones by introducing a relatively fast non-uniform flow field at the constriction and/or by aligning (focusing) the passing particles.

#### 1. Non-uniform velocity flow field

**FIG. 3.**
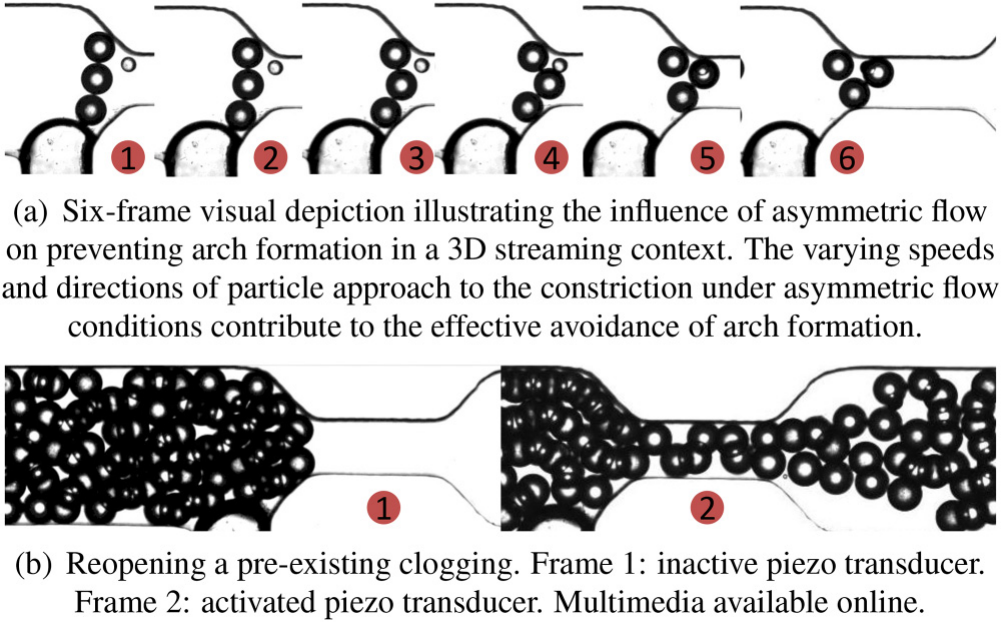
The presence of a non-uniform velocity gradient deters arch formation (a), and in the event of potential clogging, facilitates a return to an unclogged state (b). (a) Six-frame visual depiction illustrating the influence of asymmetric flow on preventing arch formation in a 3D streaming context. The varying speeds and directions of particle approach to the constriction under asymmetric flow conditions contribute to the effective avoidance of arch formation. (b) Reopening a pre-existing clogging. Frame 1: inactive piezotransducer. Frame 2: activated piezotransducer. Multimedia available online10.1063/5.0214436.1.

As illustrated in Fig. 7, the integration of microstreaming and Poiseuille flow, characterized by a mean flow rate of 12 μl/min micrometers per second, results in a markedly perturbed flow field. This perturbation is characterized by rapid non-coaxial lateral and vertical flow components, distinguishing it from the homogeneous flow observed in pure Poiseuille flow. The introduced asymmetry in flow disruption gives rise to an irregular three-dimensional flow pattern at the constriction, engendering disparate velocities that impact particle trajectories within this region. Varied velocities can influence particles to approach the constriction with differing speeds and directions, as depicted in Fig. 3(a). This phenomenon leads to a notable reduction in the likelihood of arch formation, wherein synchronized arrival at the constriction with uniform velocities is pivotal for arch formation. While the current investigation provides a qualitative overview of the impact of asymmetric flow on particle trajectories and arch formation, a more in-depth quantitative study is recommended for future research. Such an investigation would delve into the detailed effects of asymmetric flow in this region, specifically focusing on particle adherence to the constriction and the dynamics of arch formation.

Additionally, asymmetric flow proves to be proficient in dispersing and reestablishing flow through a pre-existing arch. The varying velocity gradient inherent in non-uniform flow aids in relocating obstructed particles with diverse velocities in distinct directions. This mechanism, illustrated in Fig. 3(b) (Multimedia available online), effectively restores the system to an unclogged state. Moreover, this approach can seamlessly integrate into an event-triggered operational mode, allowing the piezotransducer to remain inactively engaged. Activation of the piezoelectric component is only initiated when the control system, depicted in Fig. 2, detects a clogging event, thereby reopening the system.

#### 2. Particle alignment mechanism

**FIG. 4.**
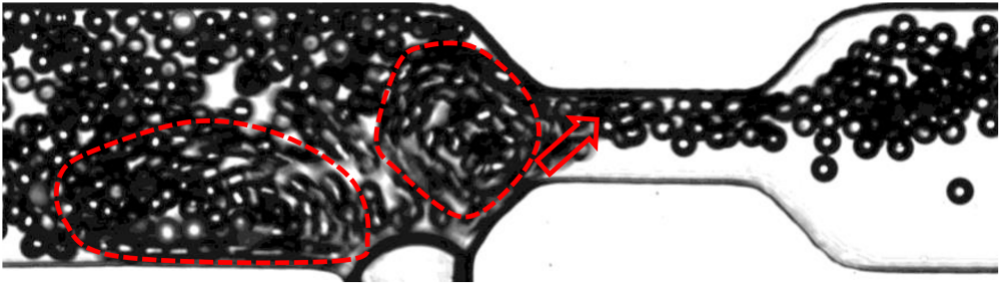
Microparticles with a W/d=3 are concentrated near the upper wall of the throat by the vortices generated by the microbubble positioned close to the throat, leaving the opposite wall free. The effective domain of the vortices is outlined with red dashed lines, and the red arrow indicates the direction of the incoming particles into the throat.

On the other hand, it has been demonstrated that microbubble streaming has the ability to align incoming particles with random lateral positions into a row. This alignment can be achieved through various operational modes, including continuous or periodic microbubble excitation. It has been shown^45^ that in a continuous combined flow of microbubble streaming and Poiseuille flow within a microchannel, particles with specific sizes undergo lateral focusing towards the center of the channel after passing through the influence of actuated microbubble(s). This suggests that the strategic placement of an actuated microbubble, even at a considerable distance from the constriction, can effectively mitigate the risk of clogging. It is important to note that a comprehensive investigation into the impact of pre-focusing the flow using a microbubble situated away from the constriction goes beyond the scope of the current study. However, within the confines of this research, our findings indicate that when the bubble is positioned in close proximity to the throat, vortices act as a guiding force for individual particles, resulting in a chain-like motion along one side of the throat. As illustrated in Fig. 4, this sequential passage of particles near the upper wall creates sufficient space near the opposite wall, reducing the probability of arch formation and subsequently mitigating the risk of particle clogging.

#### 3. Periodic mode

In situations where continuous microbubble excitation is considered unnecessary—such as when dealing with low concentrations of particles or cells, operating at very low flow rates, or when prolonged fast streaming could potentially impact cells—the Periodic Operation Mode presents an alternative strategy. This mode enables the periodic excitation of microbubbles at user-defined intervals and durations.

**FIG. 5.**
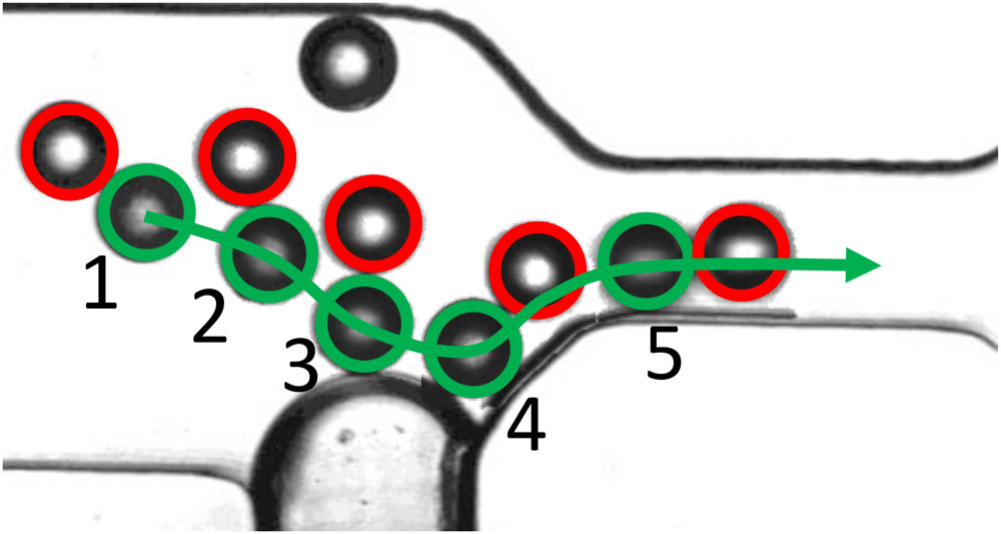
In frames 1–4, the bubble is excited, causing two particles (labeled green and red) to be drawn near the bubble’s surface. If the excitation continues, these two particles will remain in the positions depicted in frame 4 until additional particles push them toward the exit. In the current scenario, however, the microbubble is deactivated after frame 4, allowing the main flow to carry the particles to the exit through the throat.

Particles larger than the perigee—a confined region characterized by a minimal separation between the bubble’s exterior surface and the separatrix, as defined in this study for both 50 and 100 μm particles—tend to become trapped within the inner loops of microbubble streaming. This trapping occurs particularly near the microbubble’s surface and when the particle flow rate is low, persisting until either additional particles arrive or the flow rate increases enough to push them toward the exit (throat). While this phenomenon does not impede overall flow or cause clogging, it can delay particle movement. To prevent such delays, we can temporarily stop bubble excitation at strategic intervals to release the trapped particles (refer to Fig. 5). This allows the mainstream flow to sequentially transport these particles through the constriction. The pulsation of this periodic mode is adjustable based on operational conditions.

The choice between continuous or periodic operation modes depends on various factors, including particle sizes and characteristics, particle concentration, and overall flow rate. In general, continuous excitation of microbubbles is suitable for higher particle concentrations and flow rates. At the same time, adopting periodic modes becomes advantageous in preventing clustering, especially when there is a desire for particles or cells (with low repulsion) to accumulate during relatively slow flow rates. This mode serves to avert particle buildup by enabling their passage through the throat when the piezoelectric is inactive, thereby mitigating congestion in the region.

### B. Disruption and opening of particle clusters

**FIG. 6.**
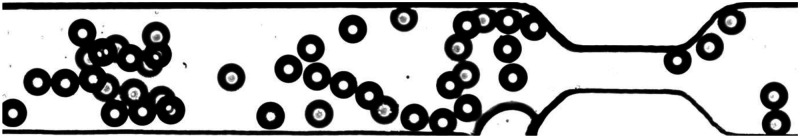
Clusters comprised of particles with the W/d=1.5 traverse the microchannel. The shear induced by microstreaming effectively breaks down these clusters into individual units or smaller subclusters, mitigating the risk of clogging.

The onset of arch formation is reliant on the proximity of particles within the microchannel. As particles traverse the channel, they frequently aggregate into clusters, leading to the formation of arch structures due to the cohesive properties inherent in these particle clusters. The presence of clusters in microfluidic devices becomes more pronounced when particles with low repulsion or cells accumulate and form aggregates on channel walls. Subsequently, these aggregates detach from the walls and re-enter the fluid. Identified as potential contributors to clogging, these clusters pose challenges in microfluidic applications and lab-on-a-chip devices. Various factors, including particle size, characteristics, concentration, fluid properties, channel geometry, and surface characteristics, contribute to the formation of clusters and subsequent microchannel clogging.^55^ Microstreaming-induced shear stresses can potentially disintegrate these clusters into individual units or smaller subclusters (see Fig. 6). The modulation of shear forces depends on factors such as microbubble size, excitation frequency and amplitude, and the characteristics of the surrounding fluid. Breaking down clusters not only reduces the risk of clogging at subsequent constrictions but also minimizes the potential for clogs throughout the entire microfluidic network in lab-on-a-chip devices, ensuring prolonged operational efficiency.

## IV. RESULTS AND DISCUSSION

This section initiates an exploration of the flow dynamics, with a specific focus on the interplay of microstreaming at the constriction zone under diverse amplitudes of microbubble excitations. Subsequent to this, we present the findings from a series of statistical experiments designed to probe the anti-clogging efficacy across different operational modes and concurrently evaluate the efficiency of microstreaming in dispersing clusters of microparticles. The previous research^12^ and our preliminary study^42^ both suggest that clogging vulnerability is not highly sensitive to changes in flow velocities. However, achieving an optimal intensity ratio between Poiseuille flow and microbubble streaming is essential for maximizing system efficiency in our anti-clogging strategy. We have chosen a Poiseuille flow rate of 12 μl/min and a microbubble excitation amplitude of 90 V (peak to peak, staying below 120 V to avoid bubble instability) to enhance the microstreaming effect at the channel’s throat. While higher flow rates are feasible, they tend to dominate Poiseuille flow and create smaller vortices, thereby reducing anti-clogging efficiency. To ensure consistency and comparability, all experiments were conducted using these parameters.

Additionally, in all experiments where the piezotransducer was activated to induce optimal microstreaming velocity, the protrusion depth of the microbubble was consistently maintained at the optimized size, equivalent to half the width of the cavity. In our specific microchip setup, the frequency of 18.4 kHz resulted in the most significant microstreaming. This frequency produced the fastest vortices that could cover the entire width of the microchannel at the given amplitude, compared to other tested frequencies with the same amplitude. Consequently, this frequency was held constant during all experiments in this study. It is important to highlight that the applicability of the technique extends beyond the utilization of PDMS microchannels, polystyrene particles, and air microbubbles. The proposed method is universally adaptable, accommodating various materials and gases to suit diverse applications.

### A. Analysis of microstreaming effects in the constriction zone

**FIG. 7.**
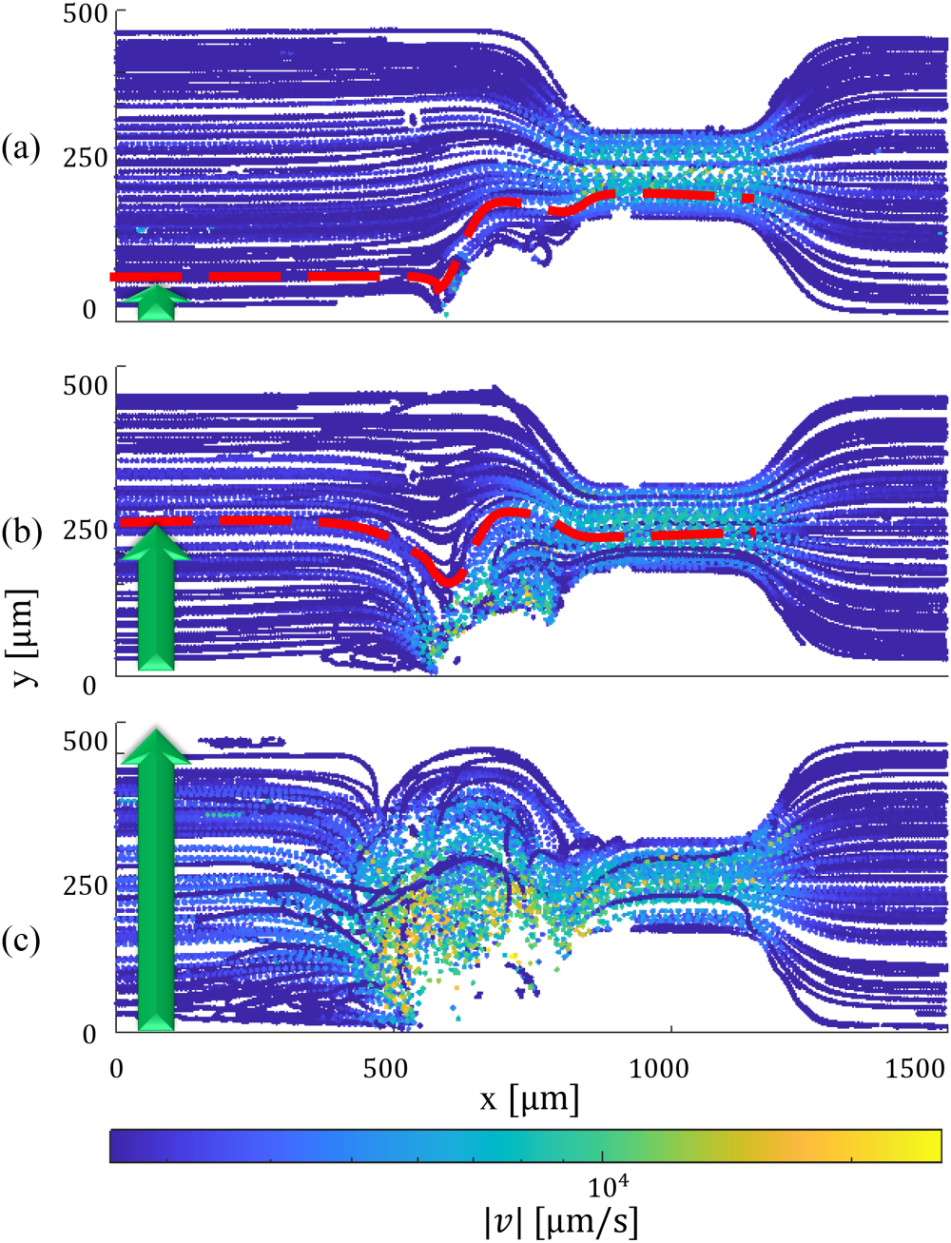
Tracking 2 μm tracer particles within the interplay of Poiseuille flow (left to right) and microbubble streaming induced by continuous excitation of the piezoelectric transducer at frequencies of 18.4 kHz near the microchannel constriction. Cases (a), (b), and (c) correspond to excitation amplitudes of 20, 60, and 90 $V_{\mathrm{pp}}$, respectively. In this examination, it is evident that case (a) exhibits the smallest vortices with minimal manipulation of incoming particles, while cases (b) and (c), with increased excitation amplitudes, lead to an elevated level of $y_{s}$ and the generation of highly disturbed 3D flow with maximum streaming velocities of 19 and 25 mm/s, respectively.

To gain a deeper understanding of the underlying mechanisms governing microbubble-induced anti-clogging effects, we conducted experiments to elucidate the interplay between microbubble streaming and Poiseuille flow near the microchannel constriction. Employing General Defocusing Particle Tracking (GDPT) analysis, as outlined by Barnkob *et al.*,^56^ we investigated this interaction under various amplitudes of piezotransducer excitation (20, 60, and 90 $V_{\mathrm{pp}}$), while maintaining a constant mean flow rate of 12 μl/min [depicted in Figs. 7(a)–7(c), respectively]. The experiments utilized 2 μm tracer particles with valid Stokes numbers, ensuring their close adherence to the streaming pattern. Applying 20 $V_{\mathrm{pp}}$ [Fig. 7(a)] revealed minimal manipulation of microparticles near the constriction. The separatrix line (red dashed line), indicating the maximum lateral level of incoming particles directed toward the core of counter-rotating vortices, remained relatively low ( $y_{s}=50\;\mu m$). Particles above this line bypassed the vortices without experiencing the fast streaming critical for breaking down clusters. Increasing the amplitude to 60 $V_{\mathrm{pp}}$ [Fig. 7(b)] resulted in a highly disturbed 3D flow, significantly enhancing streaming up to 19 mm/s near the bubble surface. The separatrix level was raised to $y_{s}=260\;\mu m$. Finally, at 90 $V_{\mathrm{pp}}$ [Fig. 7(c)], the flow became extremely disturbed, reaching a maximum streaming velocity of 25 mm/s. In this scenario, all incoming particles, regardless of their lateral position, entered the perigee—a confined region characterized by minimal separation between the bubble’s exterior surface and the separatrix. Here, particles experienced high shear in the 3D microstreaming, significantly impeding potential arch formations at the throat and breaking up potential clusters. For the remainder of the experiments in this study, we adopted the 90 $V_{\mathrm{pp}}$ amplitude to ensure that we utilized the system’s near-maximum capacity for anti-clogging performance.

### B. Operational modes

In our investigation of operational modes, we utilized solutions outlined in Sec. II A. In contrast to previous studies on clogging, our approach involves analyzing changes in particle concentration within the wide microchannel over time, rather than simply counting escaped particles. This decision is based on the recognition that the formation of arches occurs when a sufficient number of particles simultaneously reach the constriction. This phenomenon is more effectively illustrated by observing peaks in particle concentration within the temporal trend of particles passing through the microchannel. Conversely, even with a small particle-to-neck ratio, a relatively lower particle concentration may lead to extended operation periods without encountering clogging issues.

#### 1. Standby mode (off)

**FIG. 8.**
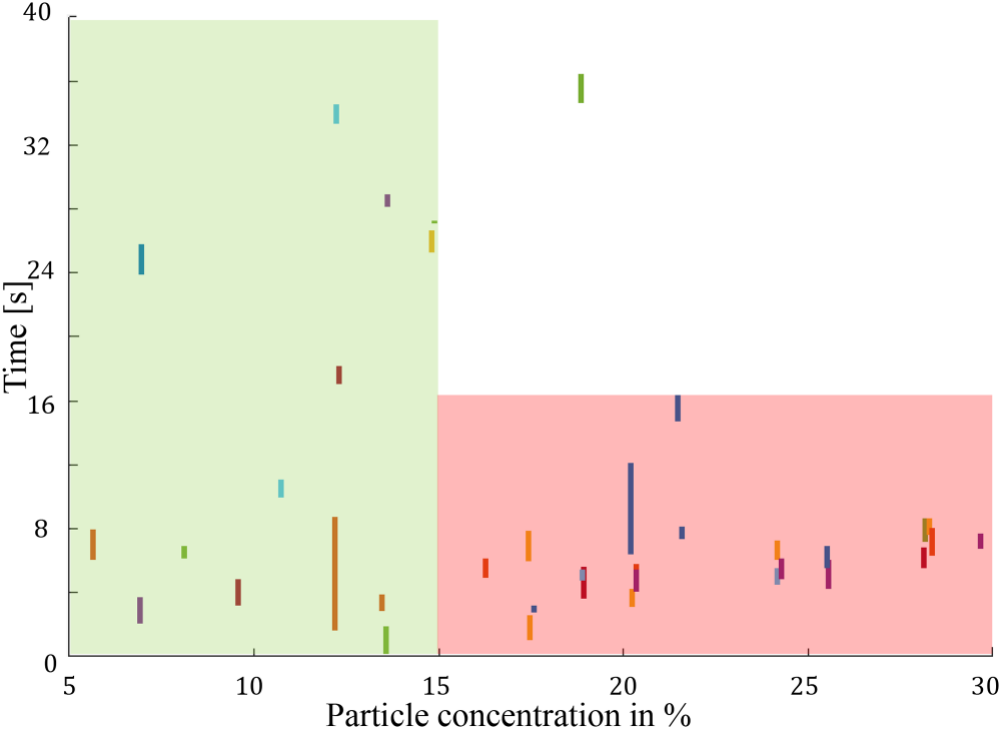
Each short vertical line represents an individual run. The horizontal position corresponds to the maximum particle concentration observed, the bottom end of the line indicates the time of this maximum, and the line’s length represents the duration until clogging. Robust clogging occurred within 16 s for runs with a particle volume fraction ( ϕ) exceeding 15%. For runs within the green rectangle, with ϕ surpassing 5%, clogging transpired within 40 s. These observations demonstrate that clogging is inevitable regardless of concentration.

To assess the likelihood of clogs in the absence of microstreaming, we conducted statistical experiments in the microchannel under standby conditions. Two types of solutions were employed—one with W/d=3 particles and another with W/d=1.5 particles. During these trials, the piezotransducer was deactivated (turned off), and only Poiseuille flow, without microbubble streaming, was utilized for microparticle transport. Notably, no clogging events were observed within the initial 200 s of the runs when using W/d=3 particles in the specified experimental conditions. Consequently, the subsequent experiments exclusively utilized the solution with W/d=1.5 particles. Figure 8 illustrates the fluctuation in particle concentration for W/d=1.5 particles across 39 experimental runs with the piezotransducer in a deactivated state. In this figure, each short vertical line represents an individual run, with the horizontal position corresponding to the maximum particle concentration observed. The bottom end of each line indicates the time at which this maximum concentration was reached, while the length of the line represents the duration until clogging occurred after reaching the peak concentration. As highlighted in the figure, robust clogging consistently occurred within 16 s from the start of the runs (with one exception) for cases where the particle volume fraction ( ϕ) exceeded 15%. In these instances, an arch formed immediately, obstructing the throat as it could not accommodate the high particle volume. Additionally, in the green rectangle, for runs with a lower maximum particle concentration but still with a particle volume fraction surpassing 5%, clogging transpired within the initial 40 s of experimentation. These observations indicate that higher particle concentrations lead to quicker clogging, while lower concentrations allow for a longer operational period before clogging occurs, showing that clogging is inevitable regardless of the concentration.

**FIG. 9.**
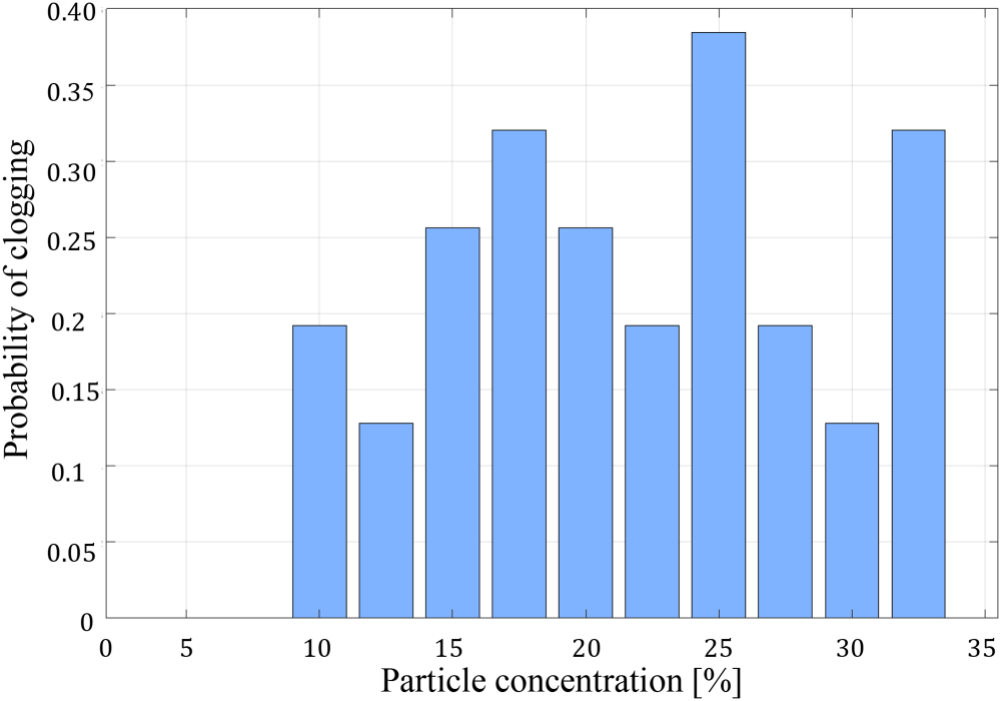
Probability distribution function (PDF) of clogging, depicting the maximum particle concentration in each of the 39 runs. The lack of a discernible correlation between particle concentration and the likelihood of clogging suggests a random nature to the clogging events in this experiment.

Figure 9 displays the probability distribution function (PDF) of clogging based on the maximum particle concentration in each of the 39 runs. The absence of a discernible correlation between particle concentration and the likelihood of clogging suggests a random nature to the clogging events in this experiment. In this experimental setup, the continuous and uninterrupted flow was only observed in cases where the particle volume fraction was 2% or lower, specifically after approximately 10 000 particles had passed through the constriction.

#### 2. Continuous operation mode

**FIG. 10.**
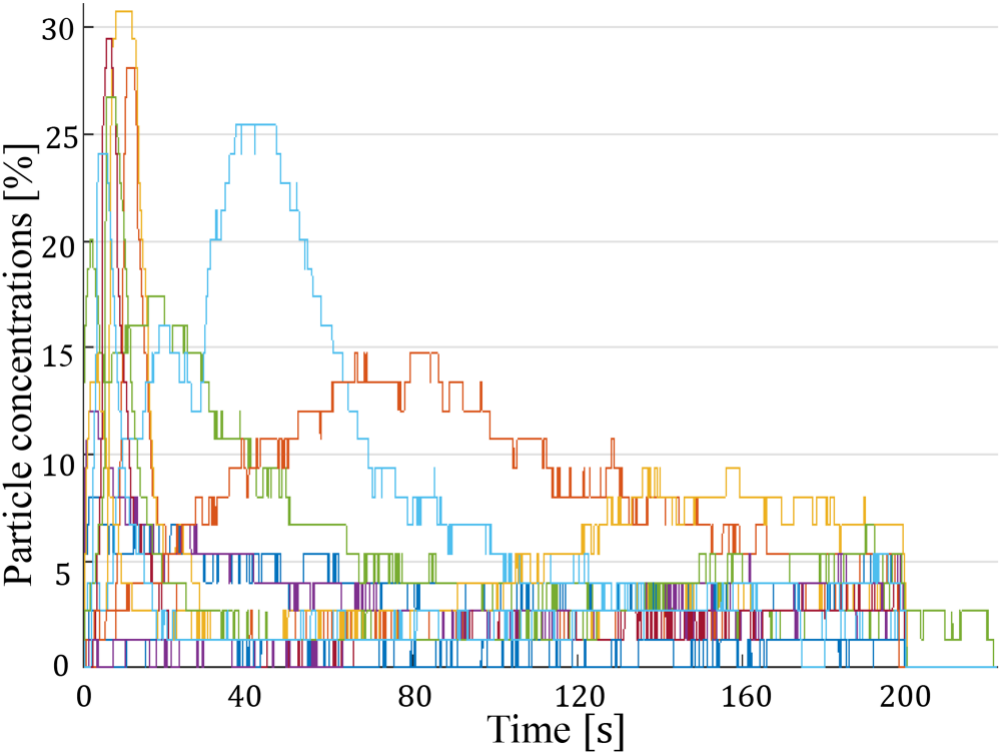
Illustration depicting particle concentration variations in 39 consecutive runs during continuous microbubble excitation. Notably, no instances of clogging were observed throughout the nearly 200 s of operation for each run.

To examine the impact of continuous microbubble excitation on the occurrence of clogs, we performed statistical experiments involving continuous flow with varying particle concentrations. Throughout these trials, the piezotransducer consistently generated 3D fast counter-rotating vortices that interacted with Poiseuille flow, disrupting potential arch formations of microparticles at the constriction. As depicted in Fig. 10, the graph illustrates significant concentration variations across 39 runs, with some cases reaching up to approximately ϕ≈30% of particle volume fraction. Notably, there were no instances of clogging observed throughout the nearly 200 s of operation for each run, indicating the robust effectiveness of this technique in preventing clogs in microchannels during prolonged operations.

Moreover, to explore the potential of continuous microbubble excitation in resolving clogging issues that had occurred during standby mode (off mode, see Fig. 8), an additional 10 runs were initiated. Remarkably, in all cases, cloggings were alleviated after the onset of piezoelectric excitation, introducing microstreaming to the system. These robust findings, demonstrating both the prevention and resolution of clogging through continuous excitation, suggest a highly effective and easily applicable approach for addressing a diverse range of clogging challenges in microfluidic applications. This leverages the biocompatible phenomenon of microbubble streaming, presenting a promising solution to clogging issues in microchannels.

#### 3. Periodic operation mode

**FIG. 11.**
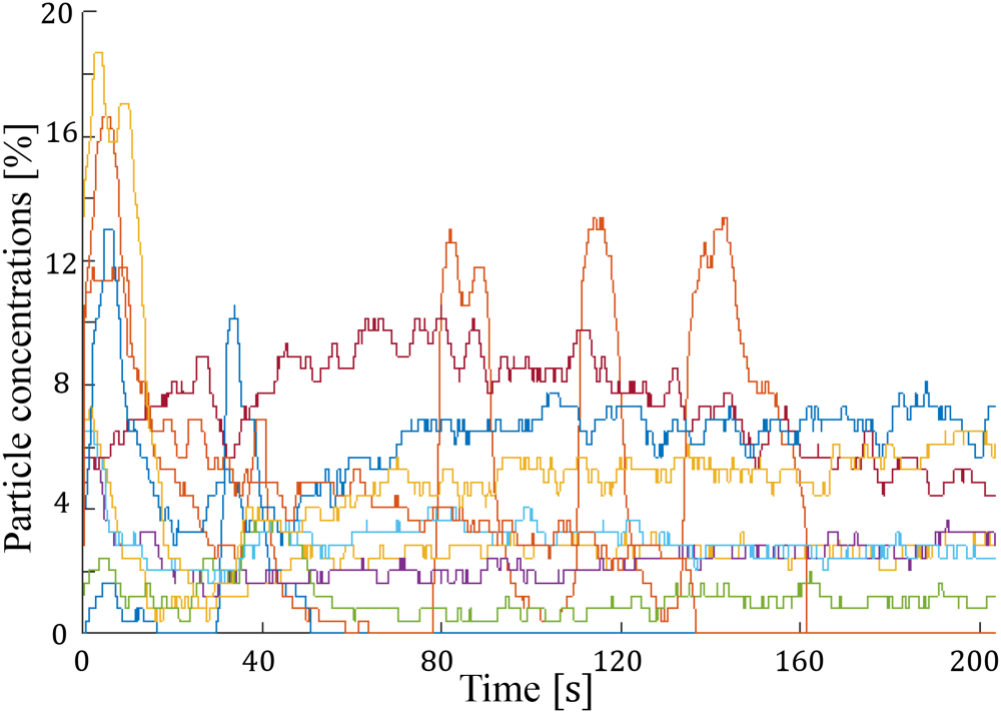
Particle concentration profiles over 10 runs, depicting the impact of periodic activation of the piezotransducer with a pulse frequency (P) of 0.250 pulses per second and excitation duration (E) of 0.125 s. Noteworthy is the absence of clogging across all cases during the 200 s duration of each run. It is noteworthy that the waves of high particle concentrations at approximately 80, 110, and 140 s might be caused by particles clustering or clogging and then re-entering the main flow upstream of the field of view (FOV).

Figure 11 illustrates the anti-clogging efficiency observed over 10 runs during the periodic activation of the piezotransducer (with a pulse per second, P, of 0.250 and excitation duration, E, of 0.125 s). Notably, no instances of clogging were observed in any of the cases during the 200 s duration of each run. This operational mode can be tailored to prevent clogging under specific operational conditions, particularly in scenarios with low flow rates, where there is a risk of particle congestion in the constriction zone. By allowing sufficient time for particles to pass through the constriction, this approach proves effective in mitigating clogging concerns.

### C. Anti-cluster

**FIG. 12.**
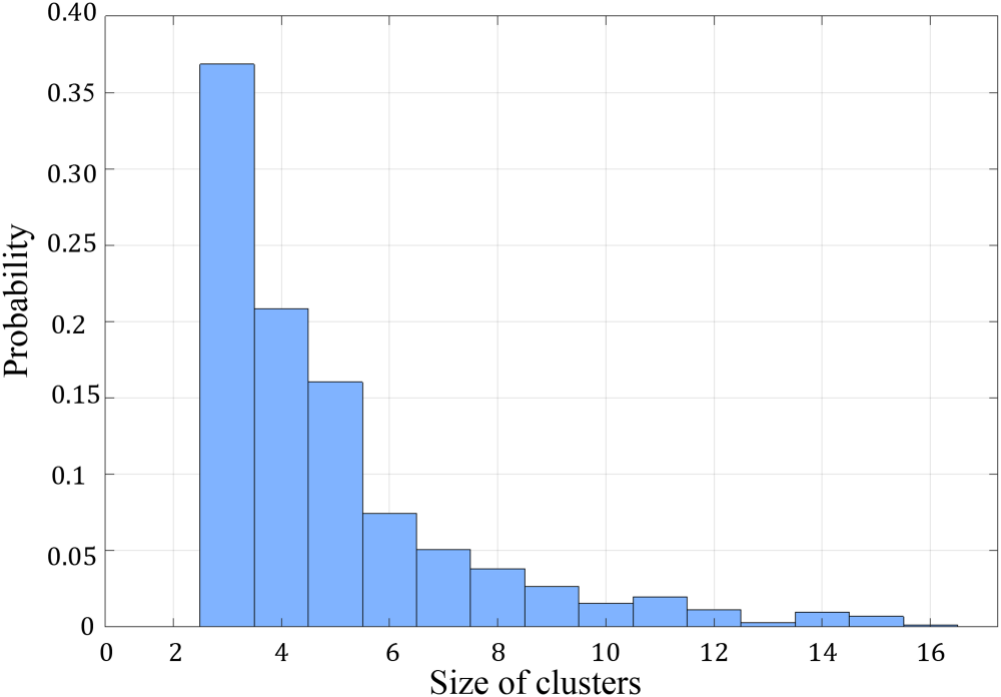
Probability distribution function depicting the passage of 1000 clusters (size 3–16 microparticles) through the microchannel, encountering microstreaming under continuous excitation of the microbubble over a 540 s period.

To investigate the effects of microstreaming on microparticle clusters, a set of experiments was performed using particles with a W/d=1.5 ratio and by adding 1 mol/l sodium chloride to the solution. The introduction of salt ions led to the encapsulation of charged groups on the particles’ surfaces. This shielding effect diminished electrostatic repulsion between particles and heightened the effective particle-wall friction, particularly since PDMS walls typically carry a slight negative charge. Consequently, particles became more prone to approaching each other and also promoting aggregation and the formation of clusters on the walls, which subsequently detached from the walls and re-entered the fluid. Here, we explored clusters of varying sizes formed by different quantities of individual particles experiencing substantial shear as they traverse the rapid 3D and non-uniform microstreaming. Figure 12 depicts the probability distribution function (PDF) based on approximately 1000 clusters, each comprising 3–16 microparticles, passing through the microchannel during a continuous excitation of the microbubble over a 540 s period. The findings indicate that, under the influence of high shear from counter-rotating vortices, all clusters undergo disintegration into single particles or smaller subclusters in a few instances. Remarkably, no instances of clogging were observed during this process. These results underscore the notable effectiveness of microbubble streaming as a proactive strategy to mitigate unwanted clogging, particularly in scenarios where the probability of cluster formation is higher, as seen in systems handling adherent particles or cells. By considering clusterization as a key factor, we are confident that microbubble streaming can break down these clusters through the high shear stress of microbubble vortices. This process leads to the singularization of particles or cells, which naturally reduces the probability of clogging, not just at constrictions but throughout complex microfluidic systems over time, thereby ensuring prolonged and continuous operation. However, the primary focus of this paper was to demonstrate the concept near a constriction, assumed to have the highest potential for clogging events. Further research is needed to evaluate the effectiveness of our method in more complex microfluidic systems. Furthermore, this capability can be leveraged not only to tackle clogging challenges but also in applications necessitating the dispersion of clusters, facilitating the individualization of microparticles.

## V. CONCLUSIONS

Our study introduces an innovative anti-clogging method utilizing microbubble streaming to significantly enhance the real-time and continuous microfluidic operations of lab-on-a-chip devices. By strategically employing microbubble streaming near constrictions and exploring diverse operational strategies—event-triggered, continuous, and periodic activation of microbubble streaming—we have demonstrated the efficacy of our approach in preventing clogging incidents and alleviating blockages as they arise. The disintegration of clusters, the mechanism facilitating particle alignment, rapid three-dimensional non-uniform flow, and its disruptive capability underscore the notable effectiveness of microbubble streaming. This is especially significant in situations where there is a heightened probability of cluster formation, as observed in systems managing adherent particles or cells.

Moreover, our investigation encompasses rigorous experimental validation, a detailed exploration of relevant phenomena, and comprehensive statistical analyses considering various scenarios, including different particle sizes and operational modes. Importantly, the inherent simplicity and independence from moving parts in our inexpensive design and concept contribute to its robustness, eliminating the need for special maintenance or cleaning. This quality makes our microchip easily integrable into diverse microfluidic applications.

Additionally, the development of a flexible control system allows our method to operate in event-triggered, continuous, or periodic modes, ensuring adaptability to a wide range of lab-on-a-chip applications and enhancing the overall seamless functionality of microfluidic systems. In summary, our innovative anti-clogging approach not only addresses clogging challenges but also presents a versatile solution applicable to dispersion scenarios, facilitating the individualization of microparticles.

Future research will focus on a comprehensive investigation of the clogging behavior of polydisperse suspensions, including different particle types such as non-spherical and biological cells, with detailed statistical analysis. This will allow us to compare the clogging dynamics of polydisperse suspensions with monodisperse suspensions under various microbubble activation conditions, considering the influence of particle size ratios relative to the throat size. Additionally, we aim to develop and optimize long-term strategies for clog prevention in microfluidic systems, specifically focusing on separating critically larger particles before they reach critical constriction points. By utilizing microbubble streaming to create effective particle separation techniques,^57^ we can ensure sustained clog-free operation by redirecting larger particles through side channels before they cause blockages. These future research directions will extend the applicability and effectiveness of our anti-clogging method in more complex and practical microfluidic applications.

## SUPPLEMENTARY MATERIAL

See supplementary material for detailed analysis of channel design and microbubble configurations: Supplementary data and figures.

## Data Availability

The data that support the findings of this study are available from the corresponding author upon reasonable request.
